# Editorial: The role of expectations on treatment outcomes: from the experimental context to the clinical practice

**DOI:** 10.3389/fpsyg.2025.1585791

**Published:** 2025-03-27

**Authors:** Eleonora Maria Camerone, Giacomo Rossettini, Joel E. Bialosky, Elisa Carlino

**Affiliations:** ^1^Nuffield Department of Clinical Neuroscience, University of Oxford, Oxford, United Kingdom; ^2^School of Physiotherapy, University of Verona, Verona, Italy; ^3^Department of Physiotherapy, Faculty of Sport Sciences, Universidad Europea de Madrid, Villaviciosa de Odón, Spain; ^4^Department of Physical Therapy, University of Florida, Gainesville, FL, United States; ^5^Brooks-PHHP Research Collaboration, Gainesville, FL, United States; ^6^Department of Neuroscience, University of Turin Medical School, Turin, Italy

**Keywords:** placebo, expectations, treatment outcomes, open-label placebo, beliefs

Expectations play a critical role in shaping the outcomes of medical treatments, influencing patient experiences and therapeutic effectiveness. Whether expectations arise consciously or subconsciously, they can act as powerful modulators of treatment responses (Carlino et al., 2016, 2012; Rossettini et al., 2023). This Research Topic, The Role of Expectations on Treatment Outcomes: From the Experimental Context to the Clinical Practice, gathers seven distinct contributions that explore how treatment expectations can affect health outcomes across different settings—from experimental paradigms to real-world clinical practices.

The first contribution by Predatu et al. examines how response expectancy impacts the efficacy of gratitude interventions in a randomized controlled trial. This study emphasizes that the effects of optimism on treatment outcomes are moderated by different levels of expectancy, particularly in enhancing positive emotions. The findings provide significant insights into how psychological interventions like gratitude journaling can be optimized through the careful management of response expectations, making this study relevant for both experimental psychologists and clinicians looking to enhance patient wellbeing through non-pharmacological means.

Moving to the clinical setting, Wessels et al. focus on the role of open-label placebo (OLP) in reducing preoperative anxiety in patients undergoing gynecological laparoscopic surgery. The Preoperative Anxiolysis and Treatment Expectation (PATE) trial explores how openly administered placebos, alongside positive expectation-enhancing videos, can significantly alleviate anxiety and postoperative pain. This innovative approach challenges traditional perceptions of placebo use and provides an ethical, non-deceptive option for improving surgical outcomes.

In a narrative review, Wilhelm et al. highlight the ways in which homeopathy leverages patient expectations to drive therapeutic success, despite the absence of active ingredients in its remedies. This review argues that while homeopathy operates primarily through placebo mechanisms, conventional medicine can learn from its success in managing patient expectations. By incorporating patient-centered communication styles and optimizing treatment rituals, evidence-based medicine can further enhance treatment outcomes.

The next article expands the discussion of expectations from lab and clinical settings to healthcare organizations themselves. Poulter et al. explore how neglecting patient expectations can induce nocebo effects, resulting in dissatisfaction, poor adherence, and negative clinical outcomes. They advocate for healthcare institutions to foster positive patient expectations at every stage of the care pathway. This article goes beyond the patient-provider interaction, emphasizing that an organization-wide commitment to managing expectations is essential for avoiding nocebo-induced harm.

Asan et al. contribute an observational study that examines the impact of media coverage on nocebo effects during the COVID-19 pandemic. Specifically, they investigate how reports linking the AstraZeneca vaccine to rare cases of cerebral venous sinus thrombosis (CVST) led to a surge in emergency department visits for headache complaints. This study underscores the broader societal implications of health communication and calls for greater vigilance in media reports to prevent unnecessary panic and strain on healthcare systems.

The power of observational learning (OL) in shaping treatment expectations is explored in the sixth article. Klauß et al. conduct a systematic review to examine how OL can induce placebo and nocebo effects. The findings suggest that watching others' treatment experiences can significantly modulate one's own expectations and outcomes, with implications for both clinical practice and patient education. This review highlights the need for future research on how to optimize OL to maximize therapeutic benefits while minimizing harm.

Finally, Cormack and Rossettini present an opinion piece on the influence of nocebo effects in exercise therapy for musculoskeletal (MSK) pain. They argue that clinicians often unintentionally create nocebo effects through their language and behavior, leading to reduced patient engagement with prescribed exercises. This article calls for clinicians to become more mindful of how their communication can impact patient expectations and outlines strategies to mitigate nocebo effects, thereby enhancing the efficacy of exercise-based interventions.

## Conclusions

The insights from this Research Topic suggest that clinicians, researchers, and healthcare organizations need to consider expectations as modifiable and measurable variables in treatment protocols. Managing these expectations—whether to harness placebo effects or prevent nocebo effects—has the potential to improve patient outcomes across a wide range of medical fields. As more attention is directed toward expectation-based interventions, the future of patient care will likely see a greater focus on psychological factors as a complement to traditional medical approaches. These articles collectively pave the way for innovative practices that enhance the efficacy of treatments by addressing the psychological underpinnings of patient experience.
